# Genomic Characterization of *Bacillus* sp. THPS1: A Hot Spring-Derived Species with Functional Features and Biotechnological Potential

**DOI:** 10.3390/microorganisms12122476

**Published:** 2024-12-02

**Authors:** Samuel Mwakisha Mwamburi, Sk Injamamul Islam, Nguyen Dinh-Hung, Orathai Dangsawat, Rapeewan Sowanpreecha, Luu Tang Phuc Khang, Napatsorn Montha, Phatthanaphong Therdtatha, Sefti Heza Dwinanti, Patima Permpoonpattana, Nguyen Vu Linh

**Affiliations:** 1 Kenya Marine and Fisheries Research Institute, Mombasa 80100, Kenya; mwakishasam@gmail.com; 2 Department of Fisheries and Marine Bioscience, Faculty of Biological Science and Technology, Jashore University of Science and Technology, Jashore 7408, Bangladesh; injamamulislam017@gmail.com; 3 Aquaculture Pathology Laboratory, School of Animal & Comparative Biomedical Sciences, The University of Arizona, Tucson, AZ 85721, USA; dinhhung@arizona.edu; 4 Scientific Laboratory and Equipment Center, Office of Surat Thani Campus, Prince of Songkla University, Surat Thani Campus, Surat Thani 84000, Thailand; orathai.d@psu.ac.th; 5 Department of Agricultural Science and Technology, Faculty of Innovative Agriculture, Fisheries and Food, Prince of Songkla University, Surat Thani Campus, Surat Thani 84000, Thailand; rapeewan.s@psu.ac.th; 6 Department of Animal and Aquatic Sciences, Faculty of Agriculture, Chiang Mai University, Chiang Mai 50200, Thailand; ltpkhcmue@gmail.com (L.T.P.K.); napatsorn.m@cmu.ac.th (N.M.); 7 Division of Biotechnology, School of Agro-Industry, Faculty of Agro-Industry, Chiang Mai University, Chiang Mai 50100, Thailand; phatthanaphong.th@cmu.ac.th; 8 Department of Aquaculture, Faculty of Agriculture, Sriwijaya University, Inderalaya 30662, Indonesia; sefti.heza@unsri.ac.id

**Keywords:** aquaculture, *Bacillus* sp., genomic adaptation, pangenome analysis, sporulation genes

## Abstract

*Bacillus* sp. THPS1 is a novel strain isolated from a high-temperature hot spring in Thailand, exhibiting distinctive genomic features that enable adaptation to an extreme environment. This study aimed to characterize the genomic and functional attributes of *Bacillus* sp. THPS1 to understand its adaptation strategies and evaluate its potential for biotechnological applications. The draft genome is 5.38 Mbp with a GC content of 35.67%, encoding 5606 genes, including those linked to stress response and sporulation, which are essential for survival in high-temperature conditions. Phylogenetic analysis and average nucleotide identity (ANI) values confirmed its classification as a distinct species within the *Bacillus* genus. Pangenome analysis involving 19 others closely related thermophilic *Bacillus* species identified 1888 singleton genes associated with heat resistance, sporulation, and specialized metabolism, suggesting adaptation to nutrient-deficient, high-temperature environments. Genomic analysis revealed 12 biosynthetic gene clusters (BGCs), including those for polyketides and non-ribosomal peptides, highlighting its potential for synthesizing secondary metabolites that may facilitate its adaptation. Additionally, the presence of three *Siphoviridae* phage regions and 96 mobile genetic elements (MGEs) suggests significant genomic plasticity, whereas the existence of five CRISPR arrays implies an advanced defense mechanism against phage infections, contributing to genomic stability. The distinctive genomic features and functional capacities of *Bacillus* sp. THPS1 make it a promising candidate for biotechnological applications, particularly in the production of heat-stable enzymes and the development of resilient bioformulations.

## 1. Introduction

The genus *Bacillus* encompasses a diverse group of Gram-positive, rod-shaped, endospore-forming bacteria that have captivated scientific interest for decades [1]. These microorganisms are ubiquitous, inhabiting a wide range of environments, including soil, water, and the gastrointestinal tracts of various organisms [2]. The remarkable adaptability and metabolic versatility of *Bacillus* species have made them subjects of intense study in fields ranging from molecular biology and genetics to biotechnology and medicine.

*Bacillus* species play multifaceted roles across different domains of life. In agriculture, many strains function as plant growth-promoting rhizobacteria, enhancing crop yields through mechanisms such as nitrogen fixation and phosphate solubilization [3,4]. Some species, such as *Bacillus thuringiensis*, are widely employed as biopesticides, offering eco-friendly alternatives to chemical pesticides [5,6]. In industry, *Bacillus* strains are workhorses for producing enzymes, antibiotics, and other valuable metabolites, with their protein secretion capabilities making them ideal for commercial applications [7,8].

The medical and biotechnological significance of *Bacillus* species is equally noteworthy. Certain strains are utilized as probiotics, contributing to gut health and potentially offering protection against pathogens [9]. The spore-forming ability of these bacteria also makes them attractive for developing stable bioformulations [10,11,12]. Furthermore, *Bacillus subtilis* serves as a model organism for studying bacterial physiology, genetics, and cellular differentiation, providing invaluable insights into fundamental biological processes [7,13,14,15]. On the other hand, the genus *Bacillus* also includes species of clinical importance. *Bacillus anthracis*, the causative agent of anthrax, is a significant pathogen affecting both humans and animals [1,16]. *Bacillus cereus* is known for causing food poisoning and opportunistic infections in humans [17]. The pathogenicity of *Bacillus* species varies widely across different organisms. Some strains exhibit insecticidal properties in plants and are harmless to humans, whereas others can cause infections in multiple host types [16,18].

Thermophilic bacteria, particularly those of the genus *Geobacillus* (formerly classified as *Bacillus*), have garnered significant attention for their biotechnological potential. These organisms thrive at high temperatures, ranging from 45 to 75 °C, and possess versatile metabolic capabilities, making them valuable for various applications [19,20]. *Geobacillus* species have demonstrated promise in biofuel production, bioremediation, and the synthesis of industrial enzymes [20]. Certain strains of thermophilic *Bacillus* also exhibit probiotic properties, producing beneficial metabolites that may enhance the health of humans and animals [21]. The thermal stability of these bacteria further increases their appeal for applications in the food industry [21]. Additionally, research on thermophilic bacteriophages is emerging, providing insights into their ecological roles and potential biotechnological applications [22]. Ongoing genomic studies continue to expand our understanding of these organisms and their practical uses [23].

The advent of whole-genome sequencing (WGS) has revolutionized the study of *Bacillus* species, allowing for unprecedented insights into their genetic makeup, evolutionary relationships, and functional capabilities [15,24,25]. Each newly sequenced *Bacillus* genome holds the potential to reveal unique metabolic pathways, novel enzymes, antimicrobial compounds, or virulence factors. This genomic information not only deepens our fundamental understanding of bacterial biology but also opens new avenues for biotechnological applications and strategies to manage pathogenic strains. In this context, the genomic sequencing and de novo assembly of a novel *Bacillus* species present a compelling opportunity to decode its genetic architecture and uncover its metabolic potential, stress resilience mechanisms, and adaptive traits. Such comprehensive genomic insights enhance our understanding of microbial diversity and evolutionary dynamics while facilitating the discovery of novel bioactive compounds, enzymes, and pathways, thereby advancing biotechnological innovation. Furthermore, they pave the way for elucidating the species’ ecological roles and interactions within microbial communities, contributing to ecosystem health and sustainability. Accordingly, this study aimed to characterize the genomic and functional features of *Bacillus* sp. THPS1 to understand its adaptation strategies and evaluated its potential for biotechnological applications.

## 2. Materials and Methods

### 2.1. Sample Collection, Isolation, and Identification of Bacteria Strain

Sediment samples were collected from a hot spring in the Tasathorn Sub-district, Phunphin District, Surat Thani, Thailand (Latitude: 17.485970° N; Longitude: 101.732582° E), with a temperature of approximately 70 °C, a pH of about 7.9, and a total mass of dissolved solids and mineral content of approximately 1980 ppm. Samples were randomly collected at a depth of 5–10 cm, serially diluted, and plated onto nutrient agar (NA) plates (Himedia, Thane, India). Colonies exhibiting distinct morphologies (e.g., shape, irregular edges, elevated surfaces, and pigmentation) were selected for further analysis. The isolate designated as THPS1 was purified through repeated streaking and stored at −80 °C in a 15% glycerol solution for long-term preservation.

For the bacterial identification, the THPS1 strain was analyzed using 16S rDNA sequencing. Genomic DNA was extracted using the Genomic DNA Mini Kit (Geneaid Biotech Ltd., New Taipei City, Taiwan) following the manufacturer’s protocol. The 16S rDNA gene was amplified using the primers 20F (5′-GAG TTT GAT CCT GGC TCA G-3′) and 1500R (5′-GTT ACC TTG TTA CGA CTT-3′) as described previously [26]. Pairwise alignment of the 16S rDNA gene sequence was performed using the standard nucleotide BLAST tool provided by the National Center for Biotechnology Information (NCBI) database (https://www.ncbi.nlm.nih.gov; accessed 17 October 2024) to verify the bacterial identity.

### 2.2. Morphological and Biochemical Analysis

The purified isolates were screened and identified using a combination of morphological and biochemical tests. Morphological characteristics were initially examined, followed by Gram staining to determine the Gram reaction of the isolates. Motility tests were performed to assess the motility of the isolated strains. Biochemical tests included catalase and oxidase tests to evaluate the presence of these enzymes, indicating aerobic respiration capabilities. Indole production was tested to determine the ability of the strains to convert tryptophan into indole, and starch hydrolysis tests were conducted to assess the capacity of the strains to break down starch into simpler sugars [27].

### 2.3. Bacterial Spore-Forming Analysis

The bacterial isolates were cultivated in Luria–Bertani (LB) broth and incubated at 37 °C for 24 h. After incubation, the bacterial cultures were overlaid onto Difco Sporulation Medium (DSM, Himedia) and incubated at 30 °C for 3 days. The bacterial suspensions were then subjected to a total plate count analysis after heating at 80 °C for 30 min. Spore efficiency was assessed by counting viable cells on nutrient agar (NA) plates. To confirm spore formation, the spore-forming bacteria were examined under a microscope at 100× magnification.

(1) $$Spore\;efficiency\;(\%)=\frac{the\;number\;of\;cell\;heat\;treatment\;(CFU/mL)}{The\;initial\;cell\;count\;(CFU/mL)}\times 100$$

### 2.4. Tolerance of Isolates to Acids and Bile Salts

The isolates were cultured in a 0.1% (*w*/*v*) pepsin saline solution at pH levels of 2.0, 3.0, and 4.0 for 3 h, and in bile salt solutions with concentrations of 1, 2.5, and 5.0% (*w*/*v*) for 3 h at 37 °C in an air shaker. After incubation, each sample was spread onto NA plates and incubated at 37 °C for 24 h. Viable cells that were resistant to the pepsin or bile salt solution were then enumerated [28].

(2) $$Survial\;rate\;(\%)=\frac{the\;number\;cell\;of\;spore\;that\;were\;tested\;(log\;CFU/mL)}{The\;initial\;cell\;spore\;count\;(log\;CFU/mL)}\times 100$$

### 2.5. Hemolytic Activity

To assess hemolysis, blood agar plates containing 5% (*w*/*v*) sheep blood were prepared. Isolated strains were streaked onto the blood agar plates and incubated at 37 °C for 24 h. After incubation, the plates were examined for changes around the bacterial colonies. A clear zone surrounding the colonies indicated β-hemolysis, reflecting the complete lysis of red blood cells. A green-hued zone indicated α-hemolysis, suggesting the partial lysis of red blood cells. No change around the colonies indicated γ-hemolysis, indicating no hemolytic activity [29].

### 2.6. Antibiotic Susceptibility Testing

The antibiotic susceptibility of the isolate was evaluated using the disk diffusion method as previously described [30]. The test organisms were cultured in LB broth at 37 °C. Seven antibiotics were tested: amoxicillin (30 µg), ampicillin (10 µg), chloramphenicol (30 µg), cloxacillin (1 µg), kanamycin (30 µg), penicillin (10 µg), and tetracycline (30 µg). The isolate was spread onto Mueller Hinton Agar (MHA) plates, and antibiotic disks were placed on the inoculated plates. The plates were then incubated at 37 °C for 24 h. The inhibition zone diameters around each disk were measured, with results categorized as follows: susceptible (S) for diameters ≥20 mm, moderately susceptible (M) for diameters between 12 and 19 mm, and resistant (R) for diameters ≤12 mm.

### 2.7. Antibiofilm Activity Assay

The antibiofilm activity of the isolate was tested against four pathogenic bacteria: Staphylococcus aureus ATCC25923, Bacillus subtilis ATCC6633, Pseudomonas aeruginosa ATCC27853, and Vibrio parahaemolyticus ATCC17802. The test organisms were cultured on NA plates at 37 °C. The isolate was cultured in Erlenmeyer flasks containing 50 mL of tryptic soy broth (TSB; Himedia, India) at 37 °C in a shaking incubator at 150 rpm until reaching a bacterial density of approximately 10^8^ CFU/mL. Cells were removed from the TSB by centrifugation at 10,000× *g* for 10 min, and the cell-free supernatant (CFS) was sterilized by filtration through a 0.4 µm membrane.

A 100 µL aliquot of each pathogenic bacterial suspension (10^8^ CFU/mL) was added to 96-well microplates, followed by 100 µL of CFS. After 24 h of incubation at 37 °C, each well was washed three times with 200 µL of phosphate-buffered saline (PBS; pH 7.2). Methanol (100 µL) was added to each well and incubated for 15 min. After the methanol was removed and the samples were air-dried, 100 µL of 0.1% crystal violet solution was added to each well and incubated for 15 min. The crystal violet solution was then removed, and after that the wells were allowed to dry completely before adding 100 µL of 95% ethanol. The absorbance of each well was measured at 570 nm to quantify antibiofilm activity [31].

(3) $$Biofilm\;inhibition\;(\%)=\frac{OD\;of\;control\;sample-OD\;of\;test\;sample}{Optical\;density\;of\;control}\times 100$$

### 2.8. Storage and Preservation of THPS1 Strain

Storage experiments were conducted at temperatures of 4 °C, 25 °C, 37 °C, and 45 °C for durations of 7, 14, 30, and 60 days. After each storage period, spore suspensions were dissolved in sterile saline solution (0.85% NaCl *w*/*v*) and spread onto NA plates. The plates were then incubated at 37 °C for 24 h. The survival rate and viability of spores were determined by spread plate counting. This method allowed for the enumeration of viable spores in each sample following exposure to different storage conditions [32].

### 2.9. DNA Extraction and Whole Genome Sequencing

Genomic DNA was extracted using the phenol–chloroform extraction method [33]. Briefly, harvested bacterial cells were lysed using EDTA-saline, RNase A, and lysozyme. SDS and proteinase K were then added, followed by three rounds of phenol:chloroform:isoamyl alcohol extraction. After purification, DNA was precipitated with sodium acetate and ethanol, washed with 70% ethanol, and resuspended in nuclease-free water. The quality and quantity of the extracted DNA were evaluated using a Nanodrop ND-1000 spectrophotometer (Thermo Fisher Scientific, Waltham, MA, USA).

Short-insert library preparation and sequencing were performed at BGI-Bangkok using the proprietary DNBseq™ platform. The process involved DNA fragmentation, End repair, A-tailing, Adaptor ligation, and DNA nanoball formation through rolling circle amplification. Sequencing was conducted using BGI’s combinatorial Probe-Anchor Synthesis (cPAS) technology on a patterned nanoarray.

### 2.10. Genome Assembly, Classification, and Annotation Analysis

Raw sequencing data were filtered to remove contaminants, adapters, and low-quality sequences using SOAPnuke v2.1.8 [34]. The filter parameters applied were “-n 0.01 -l 20 -q 0.4 --adaMis 3 --outQualSys 1 –minReadLen 150”, which excluded reads matching ≥50.0% with the adapter sequence and allowed a maximum of 3 mismatched bases. Additionally, reads with a quality score <20 constituting ≥40.0% of the total reads, reads shorter than 150 bp, and reads with an N-content ≥1.0% were discarded. De novo genome assembly was performed on the clean short reads using the Read Assembly and Annotation Pipeline Tool (RAPT) v0.5.5 [35], an NCBI pipeline designed for assembling and annotating short genome sequencing reads. RAPT integrates three key components: the genome assembler SKESA [36], the taxonomic assignment tool ANI [37], and the Prokaryotic Genome Annotation Pipeline (PGAP) [38]. These tools collectively predict coding and non-coding genes de novo, including antimicrobial resistance (AMR) genes and virulence factors, based on expert-curated data such as hidden Markov models and conserved domain architectures.

For average nucleotide identity (ANI) analysis, nucleotide-level comparisons between the newly sequenced genome and other *Bacillus* genomes were conducted using the ANI method to confirm species affiliation. These *Bacillus* genomes were retrieved from the NCBI database (https://www.ncbi.nlm.nih.gov/), accessed on 25 October 2024. The Python script PyANI v0.2.722 was utilized to calculate pairwise ANI values through two distinct methods: the MUMmer [39] and the BLAST+ method [40], using the ANIm and ANIb options, respectively. Valid strains of *Bacillus* were retained for further analysis, whereas misidentified strains were excluded. A threshold value greater than 94% [41,42] was used to confirm species-level ANI similarity.

### 2.11. Taxonomic Classification

The assembled draft genome sequence data were uploaded to the Type (Strain) Genome Server (TYGS), a free bioinformatics platform accessible at https://tygs.dsmz.de (accessed on 22 May 2024), for whole genome-based taxonomic analysis [43]. This analysis utilized recent methodological updates and features [44]. Nomenclature, synonymy, and the associated taxonomic literature were provided by TYGS’s sister database, the List of Prokaryotic names withstanding in Nomenclature (LPSN), available at https://lpsn.dsmz.de (accessed on 22 May 2024) [44]. Additionally, a robust taxonomic analysis was performed using the WGS of other *Bacillus* spp. with thermophilic potential retrieved from the NCBI database, accessed on 25 October 2024. A single-copy phylogenetic tree was conducted and analyzed using OrthoFinder v3.0.1b1 [45] in Python v3.12.0.

### 2.12. Genome Annotation

Genome annotation and analysis were performed using multiple tools and databases. In addition to the annotation performed by PGAP, a further round of annotation was conducted on the assembled genome using Prokka v1.14.6 [46] for confirmation. Protein homology analysis was performed using the UniProt Swiss-Prot/TrEMBL database and the NCBI Conserved Domain database (CDD) [47], accessed on 20 June 2024.

To identify mobile genetic elements (MGE) and potential horizontal gene transfer (HGT) events, specialized tools were employed. Putative HGT events were predicted using Alien_Hunter v1.7 [48]. MGEs were detected using mobileOG-db (beatrix-1.6) [49], applying the following parameters: *e* score of 10^−5^, *k* value of 1, query score of 80, and *p* ident value of 60. Phage detection and CRISPR analysis were conducted using a combination of tools. Prophage regions were detected and annotated using Phigaro v2.3.0 [50]. Both dsDNA and ssDNA bacterial genomes (phage) were identified using VirSorter v2.2.4 [51]. CRISPR arrays and associated Cas proteins were identified using CRISPRCasFinder v4.2.20 [52].

Antibiotic resistance genes and biosynthetic gene clusters (BGCs) were analyzed using specialized databases and tools. Resistance genes were identified using the Comprehensive Antibiotic Resistance Database (CARD) with Resistance Gene Identifier (RGI) v6.0.3 [53]. BGCs were detected and classified using antiSMASH v7.1.0 (https://antismash.secondarymetabolites.org/; accessed on 16 June 2024) with default settings [54].

Virulence factor analysis was conducted using the Virulence Factor Database (VFDB) [55]. Potential virulence factors were identified by comparing predicted protein sequences against the VFDB’s dataset of experimentally verified bacterial virulence factors. BLASTP searches from NCBI databases (https://www.ncbi.nlm.nih.gov/, accessed on 20 June 2024) were performed with an E-value threshold of 10^−5^, and hits with ≥40% sequence identity and ≥50% query coverage were considered potential virulence factors. Identified virulence factors were further categorized into major functional categories, including adherence, invasion, secretion systems, and toxins, based on the VFDB classification scheme. Additionally, the VFanalyzer v2.0 tool (http://www.mgc.ac.cn/VFs/ accessed on 20 June 2024) provided by the VFDB was utilized to systematically identify known and potential virulence factors in the genome.

To annotate functional genes of isolate THPS1, the web-based BlastKOALA v1.3 tool (part of the KEGG [Kyoto Encyclopedia of Genes and Genomes] suite) was used (https://www.genome.jp/kegg/, accessed on 20 June 2024), with default parameters set to perform a BLAST search against the prokaryotic species database within KEGG. Functional orthologs (K numbers) were assigned based on sequence similarity to known proteins.

### 2.13. Comparative Genomic Analysis

We performed a pangenome analysis using the genomes of 19 *Bacillus* species, including thermophilic species. Eighteen of these genomes were obtained from GenBank assemblies based on BLAST findings during the phylogenetic tree construction, while one genome (novel) was newly sequenced for this study. The pangenome of the 19 genomes was generated using Anvi’o v8 [56], following the pangenomics workflow (https://merenlab.org/2016/11/08/pangenomics-v2/, accessed on 20 June 2024). In brief, the workflow involved executing several scripts. The “anvi-gen-contigs-database” script was utilized to create a database with Prodigal v2.6.3 [57] for identifying the open reading frame in contigs, and “anvi-run-ncbi-cogs” was employed to annotate genes using the NCBI’s Clusters of Orthologous Groups (COG) database [58]. The genome database was established using “anvi gen-genomes-storage” and “anvi-pan-genome” scripts for visualization purposes. Anvi’o employs DIAMOND [59] to assess the similarity of each amino acid sequence within every genome against all other amino acid sequences across the genomes. The resulting similarity data were clustered using the Markov Cluster Algorithm (MCL) [60] based on the amino acid sequence similarity. In addition, to analyze the pangenome dynamics, a Power Law fit [61] was applied using the ggcaller v1.3.0 in Python [62]. This analysis calculated constant variables and used of the least squares method to fit an exponential decay model to both the core genome and singleton genes.

## 3. Results

### 3.1. Morphological and Biochemical Testing

Morphological and biochemical characterization revealed distinct features of Bacillus strain THPS1. Isolate THPS1 formed yellow colonies, was identified as Gram-positive, and exhibited a bacilli shape (Table 1).

**Figure 1:**
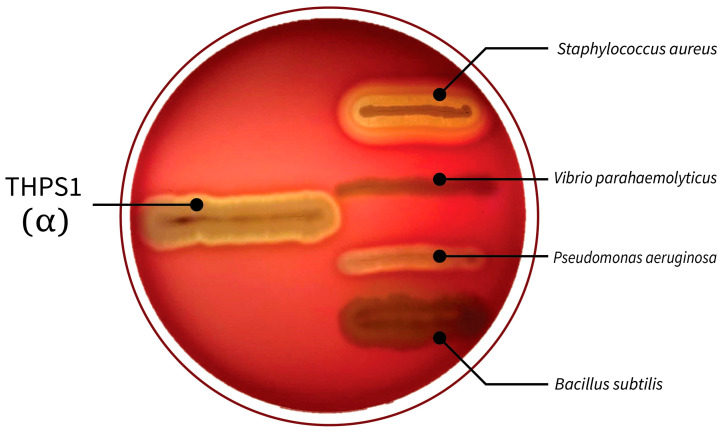
Hemolytic activity on blood agar of THPS1.

The isolate demonstrated positive results for motility, catalase activity, oxidase activity, and starch hydrolysis, indicating its ability for movement, to produce catalase and oxidase enzymes, and to hydrolyze starch into simpler sugars. Conversely, THPS1 tested negative for indole production, showing that it does not convert tryptophan into indole. Spore formation efficiency is notably high at 92.21%, suggesting a strong sporulation capacity (Table 2). Additionally, THPS1 displayed α-hemolytic activity on blood agar (Figure 1).

The isolate demonstrated survival in acidic conditions, with survival rates at different pH levels as 72.50 ± 5.29% at pH 2, 87.20 ± 6.54% at pH3, and 84.30 ± 2.09% at pH 4. Survival rates in bile salt concentrations were also evaluated, yielding the following results: 71.48 ± 1.50% at 1.0%, 69.79 ± 1.44% at 2.5%, and 68.49 ± 0.52% at 5.0% (Table 2). Regarding antibiofilm activity, the isolate exhibited the inhibition of biofilm formation for several pathogenic bacteria. The percent inhibition rates were as follows: Vibrio parahaemolyticus (70.78%), Pseudomonas aeruginosa (50.08%), Staphylococcus aureus (39.25%), and Bacillus subtilis (6.81%) (Table 2).

### 3.2. Antibiotic Susceptibility Analysis

The isolate demonstrated sensitivity to multiple antibiotics, including amoxicillin (30 µg), ampicillin (10 µg), chloramphenicol (30 µg), cloxacillin (1 µg), and kanamycin (30 µg), with inhibition zone diameters of ≥20 mm, indicating effective susceptibility. Moderate sensitivity was observed for penicillin (10 µg), with inhibition zones between 12 and 19 mm, classified as partially sensitive (Table 3).

### 3.3. Storage Stability of Bacillus sp. THPS1 Spores

**Figure 2:**
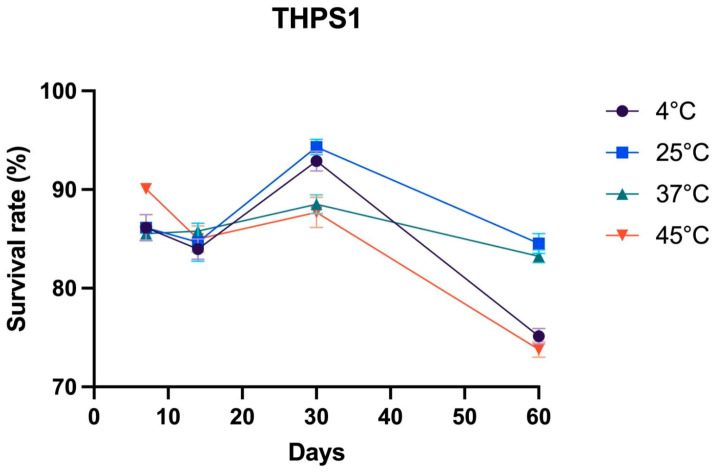
Storage stability of Bacillus sp. THPS1 spores at different temperatures over time. The survival rates (%) of Bacillus sp. THPS1 spores were measured at 4 °C, 25 °C, 37 °C, and 45 °C over storage durations of 7, 14, 30, and 60 days.

The storage stability of Bacillus sp. THPS1 spores was assessed at 4, 25, 37, and 45 °C over 7, 14, 30, and 60 days. At 4 °C, the survival rate (SR) decreased from 86.14% on day 7 to 83.96% on day 14, peaked at 92.89% on day 30, and declined sharply to 75.15% on day 60. At 25 °C, the survival rates were 86.14% on day 7, 84.67% on day 14, 94.32% on day 30, and 84.55% on day 60. At 37 °C, the SR was 84.55% on day 7, 85.78% on day 14, 88.52% on day 30, and 83.24% on day 60. At 45 °C, the SR was 90.07% on day 7, 83.33% on day 14, 87.67% on day 30, and 73.80% on day 60 (Figure 2).

### 3.4. Molecular Identification and Genomic Feature Characterization

#### 3.4.1. Molecular Identification

**Figure 3:**
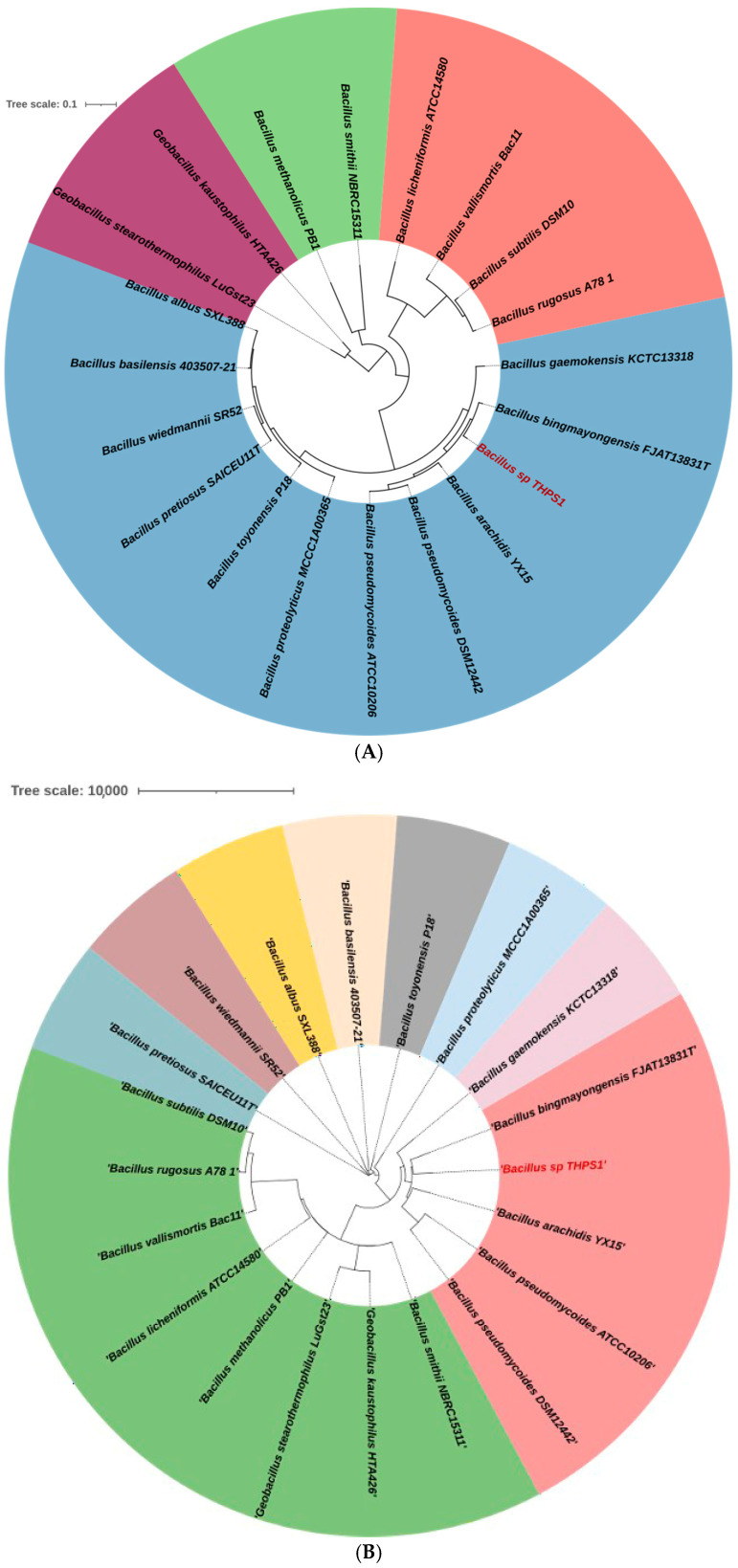
(**A**) Phylogenetic analysis based on single-copy genes of *Bacillus* sp. THPS1 and related species were performed by OrthoFinder and visualized using the ITOOL web platform (https://itol.embl.de/, accessed on 20 June 2024). OrthoFinder utilizes MAFFT v7.526 [64] for the light trimming of multiple sequence alignments to optimize runtime. FastTree v2.1 [65] was then used to construct a phylogenetic tree based on single-copy genes, providing insights into the evolutionary relationships of *Bacillus* sp. THPS1 with other *Bacillus* species and closely related thermophilic strains. (**B**) A whole-genome phylogenomic tree was constructed using RAxML v8.0.0 [66] to infer evolutionary relationships among various *Bacillus* species and related genera. The tree was visualized as a circular cladogram using ITOOL (https://itol.embl.de/, accessed on 20 June 2024). Distinct color-coded branches indicated different taxonomic groupings or species clusters. Each label corresponds to a species or strain used in the analysis, highlighting their phylogenetic positioning and comparative relationships based on whole-genome data.

The whole genome analysis of isolate THPS1 confirmed its classification within the *Bacillus* genus. Initial 16S rDNA PCR-based analysis identified THPS1 as close to *B. pseudomycoides,* based on BLAST NCBI (https://blast.ncbi.nlm.nih.gov/Blast.cgi, accessed on 18 November 2024). Additionally, a single-copy gene phylogenetic tree was constructed to support this classification (Figure 3A). However, subsequent whole genome phylogenomic tree and genomic taxonomy analysis revealed significant divergence from *B. pseudomycoides,* suggesting that THPS1 represents a novel species with the *Bacillus* genus. Further comparison using the TYGS analyzed THPS1 against *B. pseudomycoides* strains ATCC10205 and DSM12442 [63], yielding key metrics that suggested substantial genomic divergence. Specifically, the query-subject d0 similarity was 49.5%, with a confidence interval (C.I.) of 46.0% to 52.9%. The C.I. d0 metric was 28.9%, with a confidence interval of 26.5% to 31.4%. The d4 metric showed 43.3% similarity, with a confidence interval ranging from 40.3% to 46.3%. These values are significant below the threshold typically used for species identification, supporting the conclusion that THPS1 is a potential novel thermophilic *Bacillus* species (Figure 3B).

#### 3.4.2. Genomic Features

**Figure 4:**
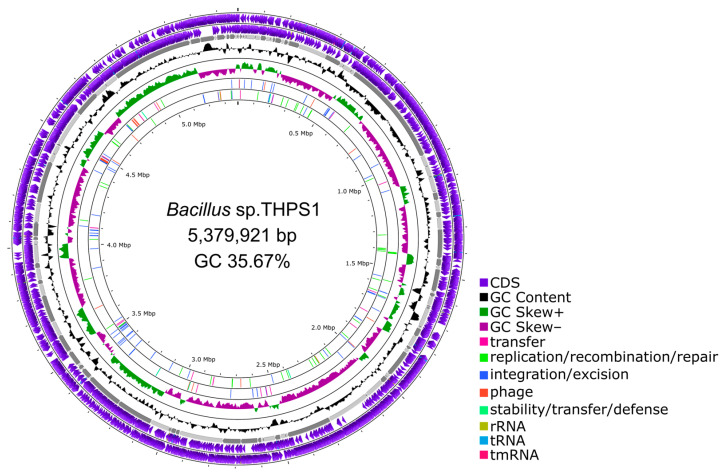
Genomic map of *Bacillus* sp. THPS1. From the outermost to the innermost circle, the map displays coding sequences (CDSs), rRNA and tRNA on the positive and negative strands, contig lengths (alternatively depicted in dark and light gray shades), GC content, and GC skew. The sixth and seventh circles illustrate the mobile genetic elements distribution on the positive and negative strands.

Genome sequencing of isolate THPS1 revealed a genome size of 5,379,921 base pairs (bp) with a GC content of 35.67%. Bioinformatic annotation identified 5606 genes on the THPS1 genome, including 13 rRNA operons comprising three copies of 23S rRNA, four copies of 16S rRNA, and five copies of 5S rRNA. Additionally, 97 transfer RNA (tRNA) genes and 5 non-coding RNA (ncRNA) genes were identified (Table 4 and Figure 4).

**Figure 5:**
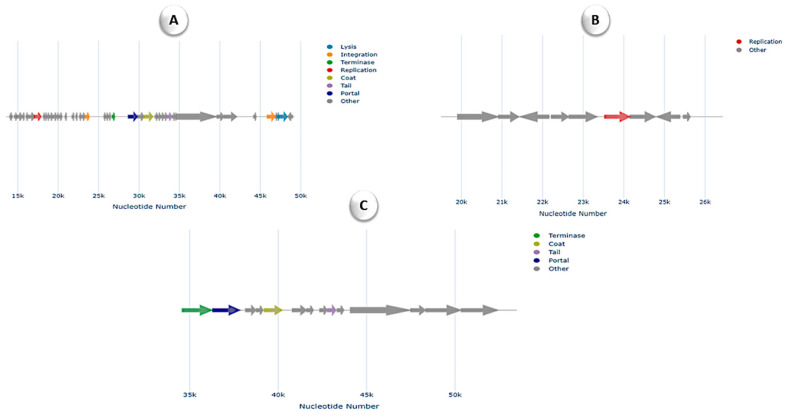
Gene arrangement of three phage regions identified in the genome, classified under the *Siphoviridae* family. (**A**) illustrates the gene structure and functional annotations, highlighting genes involved in lysis, integration, replication, coat, tail, portal, and other functions. (**B**) focuses on a specific gene cluster, showcasing replication-associated genes alongside other functional genes. (**C**) represents a phage region with a similar arrangement, emphasizing the presence of terminase, coat, tail, portal, and additional genes.

In silico analysis of the genome revealed the presence of two resistance genes associated with fosfomycin and rifamycin. Specifically, the *fosB* gene, which is confer-resistant to fosfomycin, and the *rphC* gene, associated with rifamycin resistance, were identified. Furthermore, the analysis uncovered 96 MGEs in isolate THPS1, indicating high genomic plasticity and potential for horizontal gene transfer. These MGEs were categorized into five groups: 26 involved in integration/excision (IE), 36 in replication/recombination/repair (RRR), 16 phage-related elements (P), 4 associated with stability/transfer/defense (STD), and 14 transfer-related elements (T). A total of 10 prophage regions, comprising both dsDNA and ssDNA phage elements, were identified in the genome, indicating that phages may play a significant role in shaping the organism’s genomic architecture. Among these, three distinct regions were classified under the *Siphoviridae* family, highlighting the potential contribution of phages to horizontal gene transfer, genomic variation, and the organism’s adaptation to its environment (Figure 5).

These regions displayed a variety of functional gene clusters associated with phage replication, structure, and integration. Notably, regions depicted in Figure 5A,C feature terminase, coat, tail, and portal proteins that are characteristic of *Siphoviridae* bacteriophages, which are responsible for packaging and assembling phage particles. The region shown in Figure 5B, though less complex, includes replication-related genes, further indicating the integration of these phage elements within the host genome. The presence of these phage sequences suggests the potential for horizontal gene transfer. Additionally, THPS1 contained five CRISPR/Cas features, including CRISPR arrays and Cas genes. This extensive CRISPR/Cas system suggests a robust defense against foreign genetic elements, contributing to the isolate’s genomic stability and resistance to phage infections.

**Figure 6:**
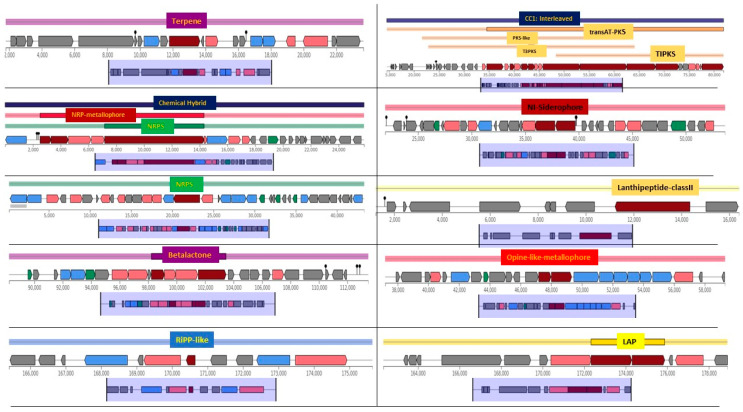
AntiSMASH analysis of the novel *Bacillus* genome identified 12 biosynthetic gene clusters (BGCs) of various types, each showing varying degrees of similarity to known clusters. This figure displays the BGCs identified in the analyzed genome, including terpenes, NRPS, chemical hybrids, β-lactones, lanthipeptides (class II), RiPP-like compounds, opine-like metallophores, siderophores, and trans-AT PKS clusters. Each BGC is color-coded to represent its classification, with core biosynthetic genes, regulatory elements, and transport genes highlighted in distinct colors. The horizontal axis denotes genomic coordinates, illustrating gene arrangements within each cluster.

Biosynthetic gene cluster profiling revealed 12 putative BGCs. Among them, a terpene cluster showed 60% similarity to bacillibactin (NRP type), while an NRPS and NRP-metallophore cluster exhibited 7% similarity to the butirosin (saccharide) cluster. Additionally, an NRPS cluster had 40% similarity to the fengycin (NRP type) cluster. A transAT-PKS cluster displayed 42% similarity to aurantinins (polyketide), and a siderophore cluster was identical (100% similarity) to petrobactin. The genome also contained two RiPP-like clusters, a lanthipeptide-class II cluster, and β-lactone, opine-like metallophore, and LAP clusters, all showing 100% similarity to known counterparts (Figure 6).

Several genes related to sporulation were detected in the draft genome of THPS1, indicating its ability to form spores, which are highly resistant structures that allow survival in extreme environmental conditions. Key genes identified include DNA translocase SpoIIIE, which is involved in chromosome partitioning during sporulation, and anti-sigma F factor antagonist and anti-sigma F factor, which regulate sporulation-specific sigma factors that are critical for initiating the process. Additionally, stage II/III/V sporulation proteins (E, D, Q, S, B, T, AH, AD) play roles in various steps of spore formation and development. Sporulation initiation inhibitor protein Soj and sporulation-control protein Spo0M are involved in regulating and initiating sporulation, respectively. Finally, sporulation proteins YpeB and YtaF contribute to spore formation and maturation. Together, these genes suggest that THPS1 has a robust ability to undergo sporulation, enhancing its adaptability and survival in challenging environments.

The analysis revealed the presence of key virulence genes in *Bacillus* sp. THPS1, including *FmvB, pdgA*, and *LOS*. *FmvB*, a magnesium-responsive outer membrane protein, facilitates nutrient acquisition and metabolic adaptation, enhancing the bacterium’s survival in host environments [67]. In addition, *pdgA* (peptidoglycan N-deacetylase) and *LOS* (lipooligosaccharide) are critical for immune modulation, enabling the pathogen to evade host immune responses [68,69]. These findings suggest that the *Bacillus* isolate is well-equipped for survival and adaptability in competitive environments.

**Figure 7:**
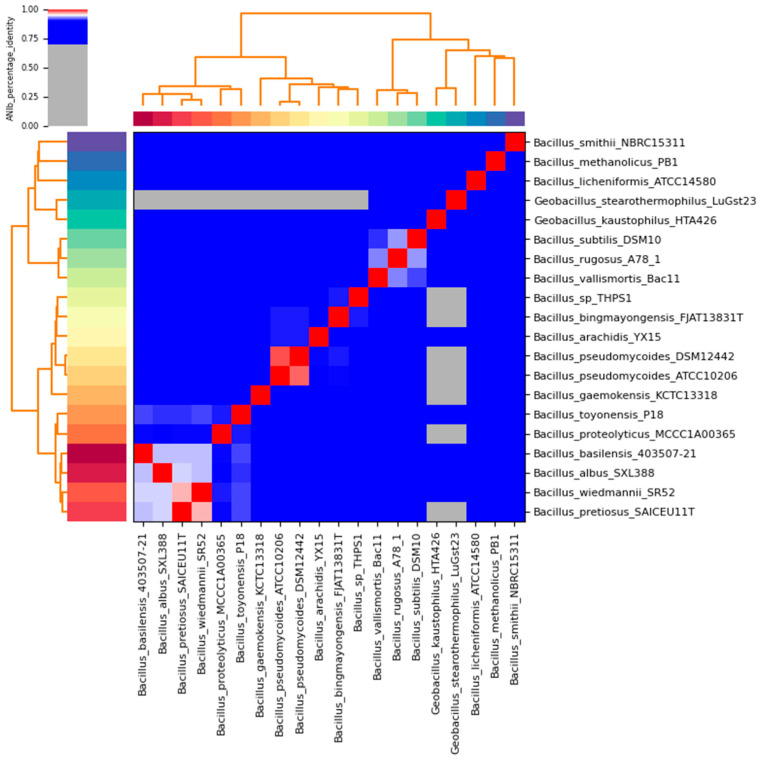
Average nucleotide identity (ANI) heatmap of *Bacillus* sp. THPS1 compared with closely related thermophilic *Bacillus* species. The ANI values range from 0.00 (low similarity, shown in blue) to 1.00 (high similarity, shown in red). The dendrogram on both axes represents the clustering of species based on their ANI values.

Additionally, the ANI heatmap demonstrated that *Bacillus* sp. THPS1 clusters more closely with *B. bingmayongensis* than with *B. pseudomycoides* and other *Bacillus* species, indicating a closer genomic similarity (Figure 7). This observation corroborated the phylogenetic analysis based on whole-genome sequencing, which contrasts with the initial 16S rDNA analysis that suggested *B. pseudomycoides*. The substantial genomic divergence from the other species supports the conclusion that *Bacillus* sp. THPS1 likely represents a distinct species within the genus, with potential thermophilic adaptations.

### 3.5. Functional Annotation

**Figure 8:**
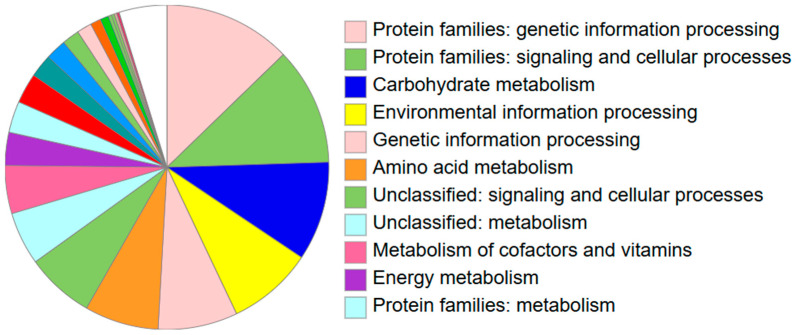
Summary of KO assignments with color coding of KEGG functional categories.

Functional annotation revealed that genetic information processing is the predominant functional category in the *Bacillus* sp. THPS1 genome, followed by significant contributions from signaling and cellular processing, carbohydrate metabolism, and environmental information processing (Figure 8). The KEGG analysis identified specific prominent signaling pathways, including the two-component system and quorum sensing. The dominance of genetic information processing underscores the isolate’s strong capabilities in managing and regulating its genetic material, which is vital for adaptation and survival in diverse environments such as hot spring soils. Additionally, prominence pathways such as glycolysis and the pentose phosphate pathway were noted under carbohydrate metabolism, highlighting the bacterium’s capacity for efficient energy production and nutrient utilization, which supports its survival and growth in nutrient-limited environments.

### 3.6. Comparative Genomics

**Figure 9:**
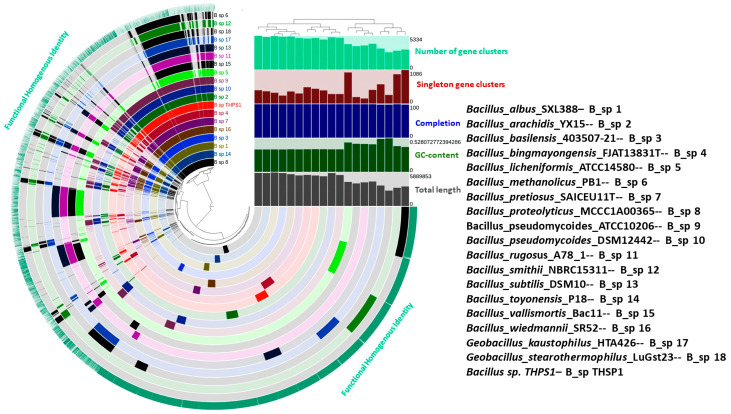
Pangenome study conducted using Anvi’o for genomes of 19 distinct *Bacillus* species, including thermophilic *Bacillus* species. The concentric rings represent functional gene clusters, including singletons and core genes. Heatmaps indicate genome metrics such as cluster counts, GC content, and genome length, highlighting both conserved and unique features across species, with *Bacillus* sp. THPS1 displaying distinctive singleton gene clusters indicative of specific adaptations.

The pangenome analysis of 19 *Bacillus* strains reveals a total of 19,366 gene clusters, of which 1888 are singleton gene clusters found in only one strain (Figure 9). The figure illustrates the distribution of core, accessory, and singleton genes across the analyzed *Bacillus* species, where *Bacillus* sp. THPS1 serves as a focal point for comparison. Core genes, conserved across all genomes, reflect essential functions shared within the *Bacillus* lineage, highlighting evolutionary stability. In contrast, accessory genes, present in only some genomes, reveal functional variability and potential adaptations tailored to specific environments or ecological niches. The presence of singleton genes in individual strains, particularly evident in *Bacillus* sp. THPS1, underscores its distinct genetic features and possible niche-specific adaptations, setting it apart within the broader *Bacillus* group. This genetic variation suggests evolutionary pressures and specialization among the species analyzed.

**Figure 10:**
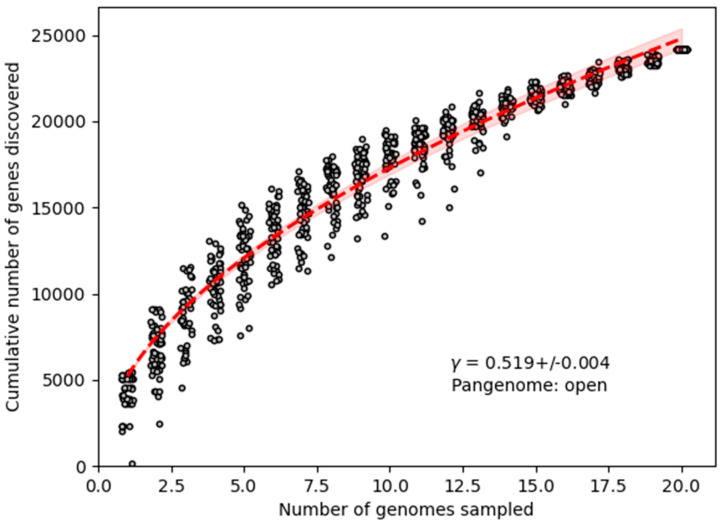
The rarefaction curve illustrates the quantity of newly identified genes resulting from random additions to a single genome. The power law fit equation is represented by γ = 0.519 ± 0.004.

Notably, the presence of a distinct set of gene clusters in *Bacillus* sp. THPS1 suggests that this strain represents a novel *Bacillus* species, further supported by its distinctive genomic characteristics. The analysis suggests an open pangenome structure (Figure 10), where additional gene clusters are likely to be found as more strains are analyzed, reflecting the species’ genomic plasticity and specialization.

## 4. Discussion

The genomic characterization and comparative analysis of *Bacillus* sp. THPS1 revealed several distinctive features that set it apart from previously characterized *Bacillus* species. Initial 16S rDNA sequencing and BLAST analysis identified THPS1 as closely related to *Bacillus pseudomycoides*. However, WGS and genomic taxonomy analysis showed substantial divergence from the *B. pseudomycoides*-type strain ATCC 7055, with key metrics, such as ANI values, falling below the species delineation thresholds. This divergence supports its classification as a novel species within the *Bacillus* genus, emphasizing the importance of WGS in taxonomic resolution, especially when initial 16S rDNA results are inconclusive [70].

*Bacillus* sp. THPS1, isolated from a high-temperature, mineral-rich hot spring environment, displayed several genetic adaptations that supported its survival in such harsh conditions. The presence of genes associated with thermotolerance, sporulation, and secondary metabolite biosynthesis aligns with previous findings on thermophilic *Bacillus* species from extreme environments [71]. This strain’s capacity to withstand temperatures up to 70 °C and alkaline pH levels of 7.9 makes it a promising candidate for industrial applications requiring heat-stable enzymes and metabolites. Genes encoding thermostable enzymes, such as proteases and lipases, suggest its potential for use in high-temperature biocatalysis for the food and detergent industries [72,73]. Furthermore, *Bacillus* sp. THPS1 exhibited high sporulation efficiency and stress resistance, supported by sporulation-related genes, including *SpoIIIE* and *Spo0M*. Storage stability analysis demonstrated the practical applicability of *Bacillus* sp. THPS1. Spores stored at 25 °C exhibited optimal medium-term viability, peaking at 94.32% survival at day 30. In contrast, both lower (4 °C) and higher (45 °C) temperatures resulted in reduced survival by day 60. These findings suggest that 25 °C storage conditions effectively maintain viability, making *Bacillus* sp. THPS1 suitable for applications requiring ambient storage without refrigeration.

This study highlights the sporulation capacity and resistance to phage infections of *Bacillus* sp. THPS1, supported by an extensive CRISPR/Cas system that enhance its stability as a bio-agent for environments where durability is essential. Additionally, the identification of 12 BGCs in *Bacillus* sp. THPS1, including siderophore clusters, NRPS, and transAT-PKS clusters, demonstrated its capacity for adapting to various environments, including thermophilic conditions [74]. The siderophore cluster, identical to petrobactin, suggests a well-developed iron acquisition mechanism, which is crucial for survival in nutrient-limited environments like hot springs. This trait, also observed in other *Bacillus* species, enhances microbial competitiveness and holds potential biotechnological applications in settings requiring efficient iron scavenging. Large-scale genomic analyses have shown that *Bacillus* species possess a diverse array of BGCs, many of which are highly conserved and play critical roles in signaling pathways affecting bacterial physiology and development [75]. The distribution of BGCs among *Bacillus* species exhibits a degree of species specificity, with certain species being rich in specific types of BGCs [76]. This diversity contributes to the ecological versatility of *Bacillus* species, allowing them to thrive in extreme conditions such as high temperatures and alkaline environments [71]. The study of BGCs in *Bacillus* has also facilitated the development of classification frameworks and the identification of conserved synteny blocks, providing valuable insights into the evolution and organization of these important genetic elements [77]. On the other hand, phenotypic antibiotic susceptibility testing was performed to validate the genomic findings. The results showed that *Bacillus* sp. THPS1 was sensitive to amoxicillin, ampicillin, chloramphenicol, cloxacillin, and kanamycin, with a zone of inhibition ≥20 mm, indicating effective susceptibility. Interestingly, despite the presence of the *fosB* gene, which is associated with fosfomycin resistance, the phenotypic susceptibility to other antibiotics did not indicate resistance. This suggests possible low expression levels or regulatory mechanisms that limit the activity of this gene under specific conditions.

Pangenome analysis of *Bacillus* sp. THPS1 alongside other *Bacillus* species revealed significant genetic diversity. The core genome shared among *Bacillus* species encodes essential functions related to cell structure, metabolism, and environmental interactions. Notably, THPS1 possesses 1888 singleton gene clusters, emphasizing its genetic divergence, which may reflect adaptations to its hot spring environment. Certain microorganisms, such as *Acidithiobacillus*, can inhabit a wide range of temperatures and pH levels due to specific genomic adaptations [78]. Hot springs also host microbes capable of hydrocarbon degradation and methane metabolism [79], offering valuable insights into microbial evolution and adaptation to extreme conditions [80]. The detection of numerous MGEs and multiple phage-related regions, including three *Siphoviridae* phage regions, suggests a dynamic genome with high plasticity. MGEs, such as transposases and integrases, facilitate horizontal gene transfer, enabling the rapid acquisition of new traits, including antibiotic resistance [81,82]. The presence of incomplete prophage regions, alongside a highly developed CRISPR/Cas system comprising five CRISPR arrays and associated Cas proteins, suggests that *Bacillus* sp. THPS1 has undergone extensive HGT while maintaining genomic stability through phage defense mechanisms. This extensive CRISPR/Cas system likely acts as a genomic barrier, limiting the integration of foreign genetic elements while allowing the acquisition of beneficial genes that enhance adaptability [83,84]. Similar findings have been reported in other thermophilic *Bacillus* species, where CRISPR/Cas systems have been implicated in the maintenance of genomic integrity under high-stress conditions [43,85]. This combination of mobile genetic elements and robust defense mechanisms highlights *THPS1*’s potential for both rapid evolution and genomic stability, making it a promising candidate for synthetic biology applications. However, future studies should include comprehensive functional assays to directly associate genetic traits with practical applications, thereby enhancing the understanding of how these contribute to industrial settings.

## 5. Conclusions

In conclusion, the comprehensive genomic and functional analysis of *Bacillus* sp. THPS1 underscores its potential as a novel thermophilic species with existing in biotechnological applications. Its capacity to produce thermostable enzymes, antimicrobial compounds, and resilient spores positions it as a valuable candidate for industries that require stability under extreme environmental conditions. Furthermore, the strain’s genomic plasticity and CRISPR-mediated phage resistance highlight its evolutionary adaptability, solidifying its appeal for synthetic biology and environmental research. These findings not only enhance our understanding of thermophilic *Bacillus* diversity but also emphasize the immense biotechnological potential of extremophiles found in hot spring ecosystems.

## Figures and Tables

**Table 1:** Morphology of bacterial isolate THPS1.

Source	Isolate	Colony Characteristics	Shape	Gram
Hot spring sediments	THPS1	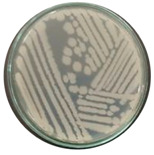	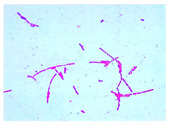	+

Note: “+” Gram-positive bacterium.

**Table 2:** Morphological and biochemical test results for isolate *Bacillus* sp. THPS1.

Parameter Analysis		Isolate THPS1
Motility		+
Catalase		+
Oxidase		+
Indole		−
Starch hydrolysis		+
Spore formation efficiency (%)		92.21
Hemolytic activity		α
Morphology	Color	Yellow
	Gram staining	+
	Shape	Bacilli
% survival rate in acids	pH 2	72.50 ± 5.29
	pH 3	87.20 ± 6.54
	pH 4	84.30 ± 2.09
% survival rate in bile salts	1.0%	71.48 ± 1.50
	2.5%	69.79 ± 1.44
	5.0%	68.49 ± 0.52
% Inhibit biofilm formation	*Vibrio parahaemolyticus*	70.78
	*Pseudomonas aeruginosa*	50.08
	*Staphylococcus aureus*	39.25
	*Bacillus subtilis*	6.81

Note: “+” positive; “−” negative

**Table 3:** Antibiotic susceptibility results for *Bacillus* THPS1.

Antibiotic	Concentration (µg/disk)	Susceptibility	Zone Diameter (mm)	Classification
Amoxicillin	30	Sensitive (S)	≥20	Effective
Ampicillin	10	Sensitive (S)	≥20	Effective
Chloramphenicol	30	Sensitive (S)	≥20	Effective
Cloxacillin	1	Sensitive (S)	≥20	Effective
Kanamycin	30	Sensitive (S)	≥20	Effective
Penicillin	10	Moderate (M)	12–19	Partial sensitivity

**Table 4:** Genomic feature distribution of the bacterial isolate in this study based on the RAPT (https://github.com/ncbi/rapt, accessed on 20 June 2024) analysis pipeline.

Features		Isolate THPS1
GenBank accession		-
Species		*Bacillus* sp.
Size (bp)		5,379,921
Minimum sequence length		223
Maximum sequence length		458,254
Contigs		144
Species		*Bacillus* sp.
L50		16
N50 (bp)		83,797
Completeness (CheckM) (%)		99.12
Contamination (%)		0.10
Genes		5606
CDSs		5492
Genes (coding)		5259
Genes (RNA)		114
rRNA	*5S*	5
	*16S*	4
	*23S*	3
tRNAs		97
ncRNAs		5
Pseudo Genes		233

Note: “-” under submission.

## Data Availability

Data are available on request from the authors.
